# G-quadruplexes and associated proteins in aging and Alzheimer’s disease

**DOI:** 10.3389/fragi.2023.1164057

**Published:** 2023-06-01

**Authors:** M. J. Vijay Kumar, Rodrigo Morales, Andrey S. Tsvetkov

**Affiliations:** ^1^ The Department of Neurology, The University of Texas McGovern Medical School at Houston, Houston, TX, United States; ^2^ Centro Integrativo de Biologia y Quimica Aplicada (CIBQA), Universidad Bernardo O’Higgins, Santiago, Chile; ^3^ The University of Texas Graduate School of Biomedical Sciences, Houston, TX, United States; ^4^ UTHealth Consortium on Aging, The University of Texas McGovern Medical School, Houston, TX, United States

**Keywords:** G-quadruplex, aging, senescence, neurodegeneration, Alzheimer’s disease

## Abstract

Aging is a prominent risk factor for many neurodegenerative disorders, such as Alzheimer’s disease (AD). Alzheimer’s disease is characterized by progressive cognitive decline, memory loss, and neuropsychiatric and behavioral symptoms, accounting for most of the reported dementia cases. This disease is now becoming a major challenge and burden on modern society, especially with the aging population. Over the last few decades, a significant understanding of the pathophysiology of AD has been gained by studying amyloid deposition, hyperphosphorylated tau, synaptic dysfunction, oxidative stress, calcium dysregulation, and neuroinflammation. This review focuses on the role of non-canonical secondary structures of DNA/RNA G-quadruplexes (G4s, G4-DNA, and G4-RNA), G4-binding proteins (G4BPs), and helicases, and their roles in aging and AD. Being critically important for cellular function, G4s are involved in the regulation of DNA and RNA processes, such as replication, transcription, translation, RNA localization, and degradation. Recent studies have also highlighted G4-DNA’s roles in inducing DNA double-strand breaks that cause genomic instability and G4-RNA’s participation in regulating stress granule formation. This review emphasizes the significance of G4s in aging processes and how their homeostatic imbalance may contribute to the pathophysiology of AD.

## Introduction

**FIGURE 1:**
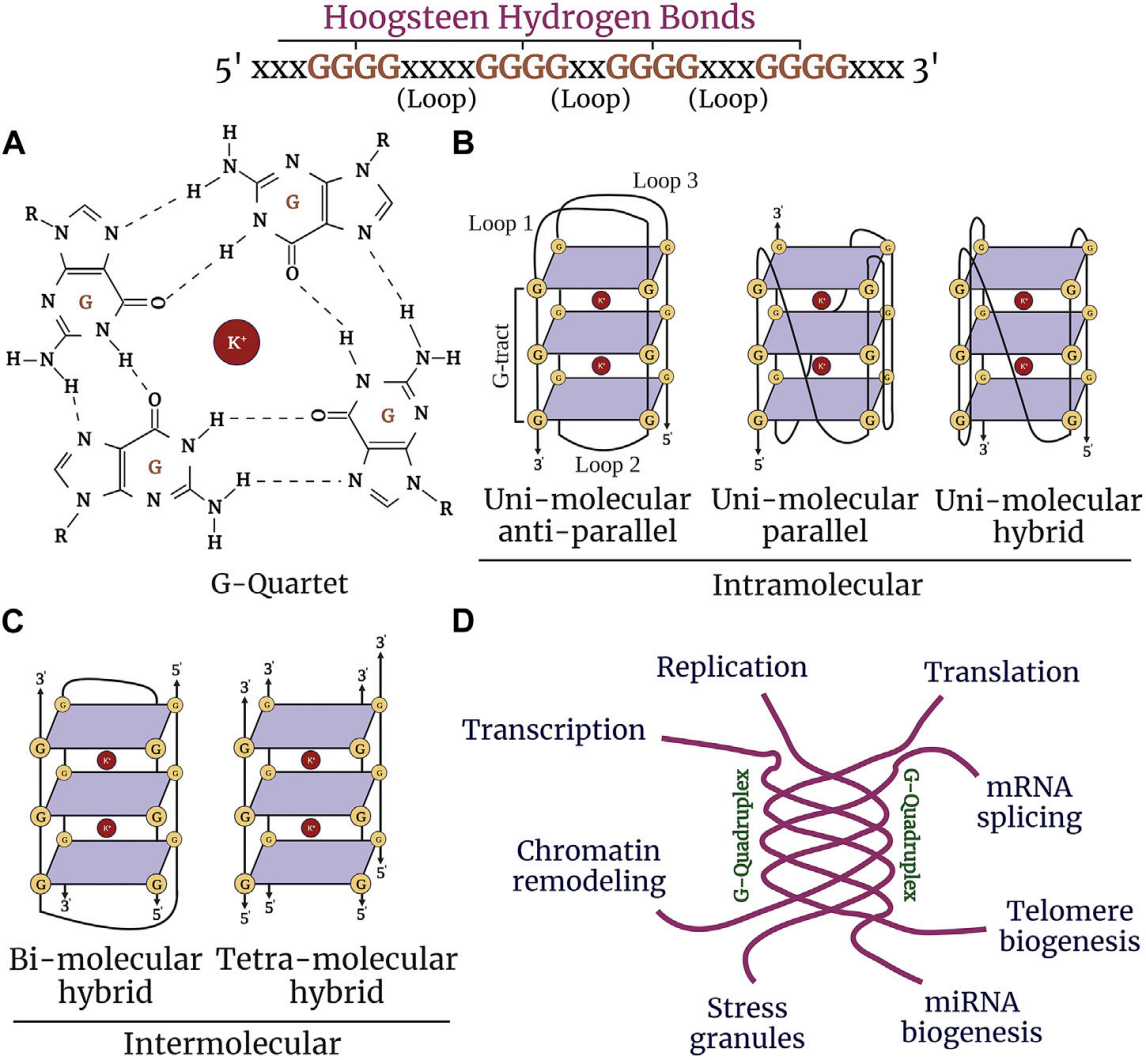
Structure and function of G-quadruplexes. **(A)** Guanine-rich nucleic acid sequences are held by Hoogsteen base-pairing to form a highly stable G-quartet structure. The stacked tetrads are stabilized by the metal ion K^+^ (highlighted in purple). **(B)** G-quadruplexes form different confirmations: intramolecular (unimolecular) parallel and anti-parallel strands, and hybrid. **(C)** Intermolecular (bimolecular and tetramolecular) quadruplexes. **(D)** G4 structures regulate nearly most of the molecular and cellular functions in the cell, such as replication, chromatin remodeling, transcription, translation, telomere maintenance, and stress granule regulation.

Alzheimer’s disease (AD) is a progressive neurodegenerative disorder that causes cerebral atrophy, cognitive decline exhibited by memory loss, and behavioral and psychiatric changes, such as depression and anxiety. Aging also involves symptoms that overlap with AD. These symptoms are associated with synaptic loss, neuronal dystrophy, vascular disintegration, and accumulation of misfolded protein aggregates (DeTure and Dickson, 2019; Long and Holtzman, 2019; Abubakar et al., 2022). Due to these insults, cells undergo multiple physiological changes and, thus, may contribute to aging and senescence traits manifested in AD. Senescent cells accumulate in aging and age-associated disorders and are coupled with cellular remodeling, which includes alterations of gene expression, transcriptional changes, and chromatin rearrangements (Di Micco et al., 2021; McHugh and Gil, 2018; Zhang et al., 2022; Kumari and Jat, 2021; van Deursen, 2014). Disease-modifying studies have focused on strategies to combat the pathological features associated with AD with the hope of either delaying the progression of the disease or reducing the severity of the symptoms (Wolfe, 2002; Godyn et al., 2016; Ramesh and Govindaraju, 2022). While significant knowledge has been gained about AD and aging, the precise mechanisms involved remain poorly understood. Interest has grown in non-canonical structures of DNA/RNA called G-quadruplexes and how these structures may initiate and propagate senescence phenotypes in age-associated neurodegenerative disorders (Antcliff et al., 2021). Guanine-rich sequences in DNA and RNA are associated with each other by Hoogsteen hydrogen bonding to form a square planar four-stranded secondary structure called a G-quartet (Lejault et al., 2021; Kim, 2019) These G-quartets stack together to form stable G4-DNA and G4-RNA structures and are stabilized by monovalent cations (K^+^>>Na^+^) (Figure 1A **)**. G4-DNA exists in different conformational states, such as intramolecular, intermolecular, and atypical structures. Diverse topological shapes were identified *in vitro* for intramolecular structures, such as parallel, antiparallel, and hybrid structures (Figures 1B,C). However, due to the constrained physiological environment *in vivo*, G4-DNA is inclined to exist and fold into a parallel conformation (Kan et al., 2007; Xue et al., 2007; Heddi and Phan, 2011; Petraccone et al., 2012; Lejault et al., 2021). More than 700,000 G4-DNA motifs were identified by high-throughput sequence analysis in human cancerous cells (Besnard et al., 2012; Biffi et al., 2013; Chambers et al., 2015). G4-DNA structures are enriched in the nucleosome-depleted and regulatory regions (e.g., promoters, telomeres, DNA replication origins), immunoglobulin heavy chain gene switch regions, and mitochondrial DNA (Besnard et al., 2012; Damas et al., 2012; Marsico et al., 2019; Tang and MacCarthy, 2021). G4-DNA is important in replication, transcription initiation, telomere maintenance, and recombination and also acts as a feedback inhibition mechanism for the initiation of replication and transcription progression (Maizels and Gray, 2013) (Figure 1D). Concomitantly, G4s in RNA are thermodynamically more stable than G4-DNA (Joachimi et al., 2009). G4-RNA structures are found in the 5′- and 3′-UTRs (untranslated regions) of the mRNA and in non-coding RNAs. G4-RNA modulates many events in RNA function, such as mRNA translocation, maturation, degradation, splicing, miRNA, PIWI-interacting RNA biogenesis, and ribosomal RNA remodeling (Darnell et al., 2001; Marcel et al., 2011; Subramanian et al., 2011; Rouskin et al., 2014; Bolduc et al., 2016; Huang et al., 2017; Kwok et al., 2018; Kharel et al., 2020a).

In recent years, the deleterious effects of G4-DNA/RNA structures on the regulation of gene expression, recombination, and genomic integrity have become the subject of intensive research. For example, overly stabilized G4-DNA structures alter transcription and promote DNA damage in neurons and glial cells (Thomas et al., 2008; Johnson et al., 2010; Noh et al., 2021). Interestingly, G4-DNA landscapes differ substantially between cell types and can have a pivotal role in cell type–specific processes (Hansel-Hertsch et al., 2016; Tabor et al., 2021). In aged cells, stabilized G4-DNA may promote DNA damage and genomic instability, thus establishing these complex structures as an exciting research objective in neurological disorders and brain aging. However, a detailed understanding of the G4 effects on brain cell function and how they contribute to developing neurodegenerative phenotypes is lacking.

In this review, we summarize recent evidence on G4-mediated regulation of key cellular processes in aging and AD and how further research on G4s could lead to the development of new avenues to design potential therapeutic strategies to alleviate age-related changes manifested in AD.

### G4-binding proteins and helicases

The homeostatic balance of G4 structures is regulated by many G4-binding proteins (G4BPs) and helicases that affect vital cellular processes, such as transcription, replication, telomere maintenance, mRNA processing, translation, and stress granule regulation (Sauer and Paeschke, 2017; Juranek and Paeschke, 2022) **(** Tables 1, 2 **)**. Telomeres are vital in genomic stability, and many proteins and helicases bind to telomeric G4 structures ensuring their stabilization and unwinding (Oulton and Harrington, 2000; Wang et al., 2011; Gardano et al., 2013; Paudel et al., 2020). Some proteins that bind to telomeric G4-DNA include POT1 (protection of telomeres 1), RPA (replication protein A), CST (CTC1-STN1-TEN1), BRCA1 (breast cancer type 1 susceptibility protein), BLM (Bloom syndrome protein), and WRN (Werner syndrome ATP-dependent helicase) (Lejault et al., 2021). Mammalian telomeric DNA is bound by a protein complex called shelterin, which prevents telomeric overhang from damage (Stewart et al., 2012; Chen, 2019). The proteins TRF1 and TRF2 (telomere repeat binding factors 1 and 2) in the shelterin complex bind to double-stranded telomeric DNA and POT1 bind to the 3′ overhang of telomere repeats and regulate the unwinding of G4-DNA structures with heterodimeric protein TPP1 (TIN2-interacting protein) (Baumann and Cech, 2001; Wang et al., 2007; Huppert et al., 2008; Hwang et al., 2012). In addition, G4 helicases WRN and BLM of the RecQ family are recruited to the telomeres and unfold the G4 structures to maintain the integrity of the telomeres and enable telomere replication (Budhathoki et al., 2014; Wu et al., 2018). WRN co-localizes with TRF2 and POT1, and WRN and BLM bind to POT1 with high affinity, indicating that telomeric DNA-binding proteins are vital for the recruitment of G4 helicases (Opresko et al., 2002; Opresko et al., 2005). Telomere-associated protein complex CST plays a key role in efficient telomere replication and maintains telomere length (Surovtseva et al., 2009; Bhattacharjee et al., 2017; Zhang et al., 2019a). CST has a strong affinity to bind to G4 structures and unwinds G4-DNA more rapidly than POT1 (Miyake et al., 2009). RPA is a single-stranded DNA-binding protein and is involved in DNA replication, repair, and recombination. RPA resolves both parallel and antiparallel G4-DNA and unwinds telomeric G4-DNA in a 5′→3′ direction (Prakash and Borgstahl, 2012). In *in vitro* assays, RPA prevents the formation of G4-DNA in the lagging strand during telomeric DNA replication (Salas et al., 2006; Nguyen et al., 2014a). BRCA1 directly interacts with telomeric G4-DNA and regulates telomerase activity and the length of telomeric 3′ overhang (Xiong et al., 2003; Ballal et al., 2009; Brazda et al., 2016). Carriers with the BRCA1 mutation have longer telomeres than non-mutation carriers (Chene et al., 2013). G4 helicase DHX36 regulates telomerase function by resolving the G4-DNA structures within the RNA component of telomerase (TERC) (Booy et al., 2012). Knockdown of DHX36 in HEK293T cells leads to reduced telomerase activity, affecting telomere length (Booy et al., 2012; Booy et al., 2015). RTEL1 (regulator of telomere elongation helicase 1) is another G4 helicase that unwinds telomeric G4-DNA in the 5′→3′ direction and is essential for telomere maintenance and DNA repair (Ding et al., 2004; Barber et al., 2008) (Table 1). In the absence of RTEL1, telomeres become short and fragile and result in the rare genetic disorder Hoyeraal–Hreidarsson syndrome (Le Guen et al., 2013).

**TABLE 1:** G4 helicases involved in a wide range of cellular functions.

G4 helicase	Affinity	Function	Comment
PIF1	G4-DNA Chisholm et al. (2012)	DNA replication Paeschke et al. (2013) and telomere maintenance Paeschke et al. (2013)	Cancer Wu et al. (2015)
BLM	G4-DNA Cheok et al. (2005)	DNA replication Rodriguez et al. (2012); Vannier et al. (2013), transcription Nguyen et al. (2014b), recombination Rodriguez et al. (2012), and telomere maintenance Vannier et al. (2013)	Bloom syndrome Bharti et al. (2013)
FANCJ	G4-DNA London et al. (2008); Lee et al. (2021)	DNA replication Wu et al. (2009)	Fanconi anemia Liu et al. (2021b)
RECQ1	G4-DNA Li et al. (2014)	Transcription Popuri et al. (2014); Lu et al. (2016) and telomere maintenance Fry and Loeb. (1999)	
WRN	G4-DNA Crabbe et al. (2004); Wu et al. (2017)	DNA replication Sarkies et al. (2012); Tang et al. (2016), transcription (Shen et al. (1998), and telomere maintenance Sarkies et al. (2012)	Werner syndrome Chai et al. (2013)
DNA2	G4-DNA (Takahama et al., 2013)	DNA replication Takahama et al. (2013) and telomere maintenance Lin et al. (2013b); Markiewicz-Potoczny et al. (2018); Kotsantis et al. (2020)	
RTEL1	G4-DNA Vannier et al. (2012)	DNA replication Wu et al. (2012), transcription Wu et al. (2012), and telomere maintenance Masuda-Sasa et al. (2008); Vannier et al. (2012)	Hoyeraal–Hreidarsson syndrome Le Guen et al. (2013)
DDX11	G4-DNA van Schie et al. (2020)	DNA replication Tippana et al. (2016)	Warsaw breakage syndrome van Schie et al. (2020)
DDX5	G4-DNA Wu et al. (2019)/G4-RNA (Dardenne et al. (2014); Herdy et al. (2018)	Transcription Wu et al. (2019) and mRNA splicing Dardenne et al. (2014)	
DHX36	G4-DNA Creacy et al. (2008; Chen et al. (2015); You et al. (2017); Ribeiro de Almeida et al. (2018)/G4-RNA (Creacy et al. (2008)	Transcription, mRNA polyadenylation Benhalevy et al. (2017), mRNA localization Maltby et al. (2020), mRNA degradation Tran et al. (2004), miRNA function Booy et al. (2014), translation Murat et al. (2018), and telomere maintenance Booy et al. (2012)	
DDX1	G4-DNA Zhang et al. (2021)/G4-RNA Zhang et al. (2019b)	IgH class switch recombination Zhang et al. (2019b)	
DHX9	G4-DNA Chakraborty and Grosse. (2011)/G4-RNA Chakraborty and Grosse. (2011)	Translation regulation Murat et al. (2018)	
DDX3X	G4-RNA Herdy et al. (2018)	Transcription Herdy et al. (2018) and rRNA remodeling Penev et al. (2019)	
DDX17	G4-RNA Dardenne et al. (2014); Herdy et al. (2018)	Transcription Herdy et al. (2018), mRNA splicing Dardenne et al. (2014), and rRNA modeling Penev et al. (2019)	
DDX21	G4-RNA McRae et al. (2017)	Translation McRae et al. (2017); McRae et al. (2020) and rRNA modeling Penev et al. (2019)	
DDX2	G4-RNA Wolfe et al. (2014)	Translation Wolfe et al. (2014)	Cancer Wolfe et al. (2014)
MOV10	G4-RNA Qureshi et al. (2012)	Translation Kenny et al. (2014)	
MOV10L	G4-RNA Qureshi et al. (2012); Vourekas et al. (2015)	piRNA biogenesis	

**TABLE 2:** G4-binding proteins involved in cellular functions.

G4-binding protein	Affinity	Function	Comment
POT1	G4-DNA	Telomere maintenance Chaires et al. (2020)	Unfolds G4s and refolds in complex with POT1-TPP1
RPA	G4-DNA	Telomere maintenance Cogoi et al. (2013)	Unfolds both parallel and antiparallel G4s
CST	G4-DNA	Telomere maintenance Bhattacharjee et al. (2017)	CTC1 contains DNA-binding site
BRCA1	G4-DNA	DNA replication and transcription Brazda et al. (2016)	
BLM	G4-DNA	DNA replication Rodriguez et al. (2012); Vannier et al. (2013), transcription Nguyen et al. (2014b), recombination Rodriguez et al. (2012), and telomere maintenance Vannier et al. (2013)	
WRN	G4-DNA	DNA replication Sarkies et al. (2012); Tang et al. (2016), transcription Shen et al. (1998), and telomere maintenance Sarkies et al. (2012)	
SP1	G4-DNA	Transcription Raiber et al. (2012)	
MAZ	G4-DNA	Transcription Soldatenkov et al. (2008); Cogoi et al. (2010); Spiegel et al. (2021)	
PARP-1	G4-DNA	Transcription Xodo et al. (2008); Cogoi et al. (2010)	Binding with c-KIT activates PARP-1
Nucleolin	G4-DNA/G4-RNA	Transcription Cogoi et al. (2014)	
hnRNP A1	G4-RNA	Telomere maintenance Kruger et al. (2010) and transcription Paramasivam et al. (2009); Goering et al. (2020)	
hnRNP H/F	G4-RNA	Transcription Decorsiere et al. (2011); Haeusler et al. (2014)	
AFF2/FMR2	G4-RNA	Transcription Yuva-Aydemir et al. (2019)	
FMRP	G4-RNA	Translation	
hTERT	G4-DNA	Telomere maintenance Paudel et al. (2020)	

FANCJ belongs to the XPD group of G4 helicases that facilitate DNA replication and recombination (Wu and Spies, 2016). It resolves G4-DNA structures in the 5′→3′ direction, and its absence leads to the persistent stalling of DNA replication at G4-DNA structures (Castillo Bosch et al., 2014). FANCJ depletion in human cells is sensitive to G4-DNA stabilization and results in elevated DNA damage and apoptosis upon exposure to G4-DNA stabilizing compound telomestatin (Wu et al., 2008). Moreover, FANCJ-deficient cells accumulate deletions at genomic sequences with G4-DNA structures, suggesting its crucial role in replication-associated DNA damage (London et al., 2008).

PIF1 is a potent G4-DNA helicase (Byrd and Raney, 2017). In yeast cells, it prevents G4-DNA–mediated genomic instability and prevents DNA double-stranded breaks (DSBs) (Ribeyre et al., 2009; Paeschke et al., 2013). Mammalian PIF1 is recruited to DNA DSB sites, promoting homologous recombination at the sequences that form G4-DNA structures (Paeschke et al., 2013). In the absence of PIF1, replication fork progression is slowed in the vicinity of putative G4-DNA motifs and increases the gross chromosomal rearrangement at G4-DNA sites (Piazza et al., 2012; Paeschke et al., 2013). G4-DNA structures at promoters are prominent binding sites for transcription factors, affecting gene expression.

SP1 (specificity protein 1) is a zinc-finger transcription factor that binds to G4-DNA structures of the *c-KIT* promoter and regulates the expression of housekeeping genes (Raiber et al., 2012). MAZ (myc-associated zinc finger) and PARP-1 (poly-ADP ribose phosphate 1) interact with G4-DNA structures upstream of the transcription start site of *KRAS* and activate *KRAS* transcription (Cogoi et al., 2010) (Table 2). Nucleolin is a nucleolar phosphoprotein involved in ribosome biogenesis, chromatin remodeling, transcriptional regulation, and apoptosis (Tajrishi et al., 2011). It selectively binds to endogenous and exogenous G-rich sequences that fold into G4-DNA and G4-RNA (Hanakahi et al., 1999). Nucleolin specifically binds to G4-hexanucleotide repeat expansion (HRE) in C9orf72 (GGGGCC)_n_ and activates molecular cascades, leading to neurodegenerative phenotypes (Haeusler et al., 2014). In hematopoietic cells, together with heterogeneous nuclear ribonucleoproteins (hnRNPs), nucleolin forms a lymphocyte-specific complex LR1 (lipopolysaccharide response factor 1) that binds to G4-DNA to form immunoglobulin heavy chain (IgH) switch regions (Dempsey et al., 1999).

G4-RNA structures are enriched in 5′-UTRs and are regulated by eIF4A linked to cancer development (Wolfe et al., 2014). DDX3X (DEAD-box helicase 3 X-linked) regulates rRNA remolding by regulating rRNA G4 structures and resolves G4-RNA at 5′-UTR of NRAS oncogene (Herdy et al., 2018; Penev et al., 2019). DDX5 and DDX17 unfold G4-RNA structures to regulate transcription and bind with hnRNPs to mediate pre-mRNA splicing (Dardenne et al., 2014; Herdy et al., 2018). DDX5 also resolves G4-DNA motifs at the Myc promoter to facilitate its transcription (Wu et al., 2019) (Table 1). G4-RNA helicase DDX21 directly binds rRNA G4 structures to regulate their functions (McRae et al., 2017). DDX21 unfolds G4-RNA structures at the 3′- and 5′-UTRs of *MAGED2*, modulating its gene expression (McRae et al., 2017; McRae et al., 2020). RNA helicase A/DHX9 (DExH-box helicase 9) binds to and resolves both G4-DNA and G4-RNA and promotes translation by unwinding 5′-UTR G4-RNA structures (Chakraborty and Grosse, 2011; Murat et al., 2018). The G4 helicase DHX36 efficiently resolves G4-RNA structures and regulates cellular processes, such as translational regulation (Murat et al., 2018; Chen et al., 2021), mRNA localization and degradation (Tran et al., 2004; Maltby et al., 2020), telomere regulation (Booy et al., 2012), long ncRNA function (Booy et al., 2016), and miRNA function (Creacy et al., 2008; Booy et al., 2014; Chen et al., 2015). DDX1, DDX24, DDX42, and DDX58 bind and resolve G4-DNA and G4-RNA structures; however, their regulatory functions remain to be characterized (Zyner et al., 2019; Zhang et al., 2021). G4-RNA helicases MOV10 and MOV10L1 are associated with different RNA regulatory pathways, and both preferably bind to G4-RNA motifs *in vivo* (Kenny et al., 2014; Vourekas et al., 2015). MOV10 binding to G4-RNA motifs is involved in FMRP-mediated translational regulation, and MOV10L1 binding is linked with piRNA biogenesis and function (Kenny et al., 2014; Vourekas et al., 2015).

Many G4-regulating proteins are linked to human diseases (Lerner and Sale, 2019; Brosh and Matson, 2020; Antcliff et al., 2021). Helicase WRN is mutated in Werner syndrome, which is characterized by accelerated aging, cardiovascular disease, and cancer (Epstein et al., 1966; Lerner and Sale, 2019). Dyskeratosis congenita, characterized by severe multisystem and bone marrow failure, is linked to mutations in RTEL1, a helicase that processes telomeric G4-DNA (Walne et al., 2013; Lerner and Sale, 2019). In Fanconi anemia, the FANCJ G4 resolving helicase is mutated and leads to cancer (Wu and Brosh, 2009; Lerner and Sale, 2019). Mutations in the helicase XPD lead to xeroderma pigmentosum and Cockayne syndrome (Lerner and Sale, 2019). Mutations in the telomere maintenance complex (the CST complex; CTC1, STN1, and TEN1) lead to severe multisystem Coats plus syndrome (Simon et al., 2016). Mutated helicase BLM causes Bloom syndrome, which is associated with cancer (Lerner and Sale, 2019). The L319P mutation in the helicase PIF1 leads to a higher risk for cancer (Wu et al., 2015). All these previously mentioned diseases, with the exception of PIF1^L319P^-linked cancer, are characterized by some degree of brain pathology and aging phenotypes. The RNA-binding protein family hnRNP is closely related to health and diseases and binds to both G4-DNA and G4-RNA structures (Geuens et al., 2016). hnRNP A1 and UP1 modulate replication, transcription, and telomere maintenance by destabilizing G4 structures and keeping them single stranded in an unfolded form (Fukuda et al., 2002; Paramasivam et al., 2009; Kruger et al., 2010; Ghosh and Singh, 2018; Clarke et al., 2021). hnRNP H/F specifically binds to unfolded G4-RNA to prevent the formation of G4 structures and its interaction with DHX36 can modulate translation (Herviou et al., 2020). hnRNP H/F plays a role in splicing and polyadenylation regulation and is associated with the *C9orf72* G-rich repeats and has been predicted to regulate RNA-processing binding to G4-RNA motifs (Decorsiere et al., 2011; Haeusler et al., 2014). Sequestration of hnRNP H might affect the expression of mRNAs containing G4-RNA motifs, and hnRNP H associates with G4-forming *C9orf72* repeats and co-localizes with G4 foci in cells derived from patients with amyotrophic lateral sclerosis (ALS) but not in non–ALS-derived cells. Formation of these G4 aggregates correlates with dysregulated gene expression in ALS patient’s brains (Prudencio et al., 2015). In ALS and frontotemporal dementia (FTD), AFF2/FMR2 regulates the expression of the *C9orf72* allele containing G-rich sequences, and knockdown of AFF2/FMR2 decreases the expression of the mutant allele, resulting in the rescue of axonal degeneration and TDP-43 pathology. Knockdown of AFF2/FMR2 also results in reduced levels of repeat RNA foci and dipeptide repeat proteins in the cortical neurons (Yuva-Aydemir et al., 2019). These findings provide insights into the mechanism underlying the toxicity and dysregulation of transcription of G-rich sequences in ALS and FTD.

Many functions of G4BPs and G4 helicases point to their importance in the regulation and modulation of G4 structures in cells. The G4 structures are implicated in various types of cancers and neurological disorders by three distinct mechanisms: i) stabilization of G4-DNA/RNA structures that cause disease, ii) abnormal de-stabilization of G4-DNA/RNA structures that cause disease, and iii) mutations that affect the expression and function of helicases and G4BPs that regulate G4 structures in cells (Tables 1, 2). These processes are interlinked as dysfunctional helicases, and G4BPs disrupt the homeostatic balance of G4 structures which may lead to senescence and progeroid phenotypes manifested in age-related neurological disorders. Therefore, a deeper understanding of the processes related to their formation, function, and recognition will be a crucial puzzle to solve to provide better insights into the regulation of G4 structures in aging and neurodegeneration.

### G4-DNA in replication

**FIGURE 2:**
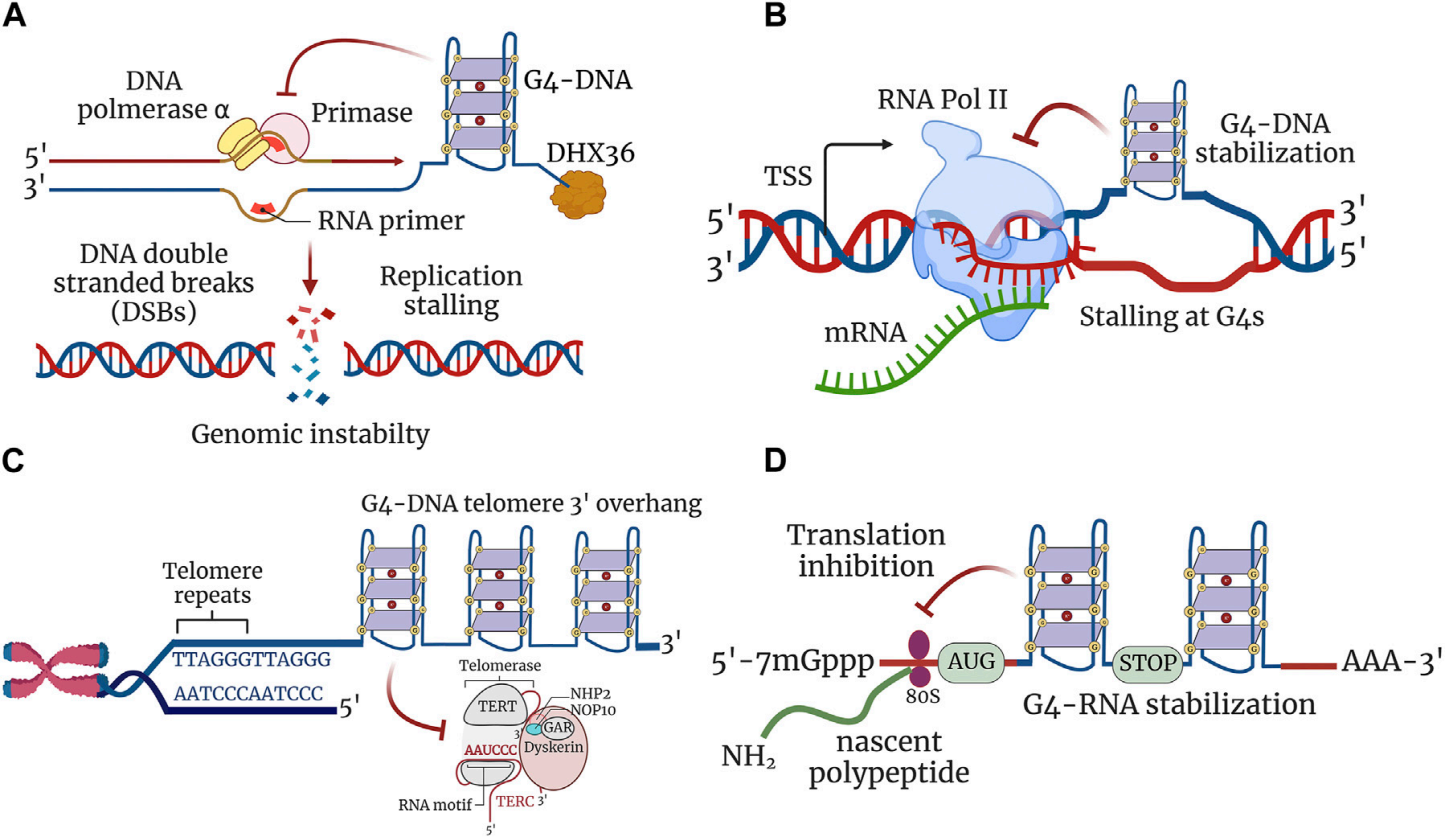
G4 structures regulate cellular processes. **(A)** G4 structures stabilized in a single-stranded DNA impede the progression of the replication fork, thereby inhibiting replication. Stabilized G4-DNA structures have to be unwound to permit the replication machinery to proceed for both leading and lagging strand synthesis. Replication stalling at G4-DNA structures leads to the formation of DNA DSBs contributing to genomic instability. **(B)** Transcription is inhibited due to stable G4-DNA structure formation downstream of transcription start site (TSS). G4-DNA structure restricts and inhibits the activity of RNA polymerase from extending the nascent mRNA and thereby stalls transcription at G4-DNA units. **(C)** G-rich 3′ overhang of telomeres forms G4 structures and are involved in telomere end protection. Stabilization of G4-DNA structures impairs telomere repeat synthesis by telomerase enzyme and leads to telomere shortening. **(D)** Formation of G4-RNA structures hinders the scanning of 5′ untranslated region (5′-UTR) by ribosomes and leads to the restriction of translation. The 80S ribosomes engaged in the translation elongation of nascent polypeptide stalled within the ORF by stabilized G4-RNA structures.

A recent genome-wide map of initiation sites of DNA replication identified G4-DNA motifs in higher eukaryotes and humans (Cayrou et al., 2011; Besnard et al., 2012; Langley et al., 2016). G4-DNA structure formation seems to be essential for the initiation of DNA replication in the cells (Valton et al., 2014) and to favor the transient opening of the double helix during DNA replication. However, once formed, these G4-DNA structures are stable and may stall DNA polymerase, thereby impeding the progression of the replication fork (Woodford et al., 1994; Weitzmann et al., 1996). Deletion of FANCJ in *Caenorhabditis elegans* results in the accumulation of DNA breaks upstream from G4-DNA (Kruisselbrink et al., 2008). Human cell lines with deletion of FANCJ accumulate DNA breaks in the vicinity of the G4-DNA structures (London et al., 2008). Therefore, these G4-DNA structures may become an obstacle to the replication machinery and interfere with the replication of both leading and lagging strands, causing DNA DSB–promoting mutagenesis, such as insertions, deletions, inversions, and recombination (Lerner and Sale, 2019) (Figure 2A). These aberrations caused by the G4-DNA structures could be significant pathogenic drivers in dividing brain cells, such as glial and endothelial cells (Lejault et al., 2020; Noh et al., 2021; Tabor et al., 2021).

To mitigate the deleterious effects of G4-DNA, G4BPs and G4 helicases specifically bind to and resolve G4-DNA structures during cell division. Eukaryotic cells have at least 10 helicases that regulate G4-DNA structures during replication: DEAH-box helicases DHX36 and DHX9; RecQ helicases BLM and WRN; Fe-S helicases FANCJ, DDX11, RTEL1, and XPD; and superfamily 1 helicases PIF1 and DNA2 (Lerner and Sale, 2019). During replication, many proteins in yeast and metazoans show high affinity and specificity for G4-DNA. These include PIF1, FANCJ, and RecQ, which play a significant role in suppressing genomic instability associated with G4-DNA formation (Fry, 2007). Disruption of PIF1 in yeast cells causes frequent stalling of DNA replication and increases gross chromosomal rearrangements proximal to G4-DNA motifs, and PIF1-deficient cells are more susceptible to the formation of DNA DSBs (Paeschke et al., 2011).

Replication stress is caused by multiple factors, such as oxidative DNA damage and aberrations in growth signaling pathways and contributes to genome instability and accelerates the aging process. RecQ helicases contribute to genome stability at stalled replication forks by activating the checkpoints, stabilizing stalled replication complexes, and preventing the formation of aberrant recombination intermediates (Bennett and Keck, 2004). In humans, mutations in WRN RecQ helicase cause genome instability and the premature aging syndrome Werner syndrome (Bachrati and Hickson, 2003). Defects in other RecQ helicases, such as BLM and RECQL4, also lead to genomic instability and premature aging syndromes, such as Bloom and Rothmund-Thomson syndromes (Larizza et al., 2006). Replication stress–induced DNA damage is detected in pre-neoplastic lesions in humans and mice, strongly implicating replication stress in the etiology of age-related diseases. DNA damage was specifically detected at fragile chromosomal sites, which were induced in cultured cells treated by drugs to inhibit DNA replication (Gorgoulis et al., 2005). Replication deficits have also been reported in AD. One study that investigated the correlation between disease pathology and replication suggests that neurons degenerate due to lethal cell-cycle defects (Yang et al., 2001). This study used fluorescent *in situ* hybridization to explore the chromosomal component of interphase neuronal nuclei in the adult human brain and found direct evidence for attempted cell cycling in AD neurons, which completes a nearly full S phase but does not initiate mitosis, thus resulting in tetraploidy. This genetic imbalance was proposed to cause a neuronal loss in AD and has provided evidence for DNA replication in affected neurons, suggesting that adult neurons attempt to divide but do not complete the process (Yang et al., 2001). Aneuploidy and DNA replication also have a role in AD pathology. In AD, some neurons re-enter the cell cycle and pass through functional interphase with complete DNA replication, which is potentially associated with neuronal cell death (Mosch et al., 2007). These crucial findings suggest that replication stress–induced genomic instability contributes to pathogenic pathways and leads to neurodegeneration in AD and aging. Replication stress could be a key element in explaining the ectopic cell-cycle events and genomic instabilities in AD. The DNA replication stress hypothesis in AD suggests that chromosomal reduplication without proper cell-cycle completion and mitotic division causes neuronal cell dysfunction and death (Yurov et al., 2011). However, this theory requires more input and research to explain the cause and consequences of genomic instability in the AD brain. Investigating G4-induced replication stress can contribute to the understanding of the course and classification of the sequences of abnormal events that generate pathologic cellular and organism phenotypes in age-related disorders, such as AD.

### G4-DNA and chromatin remodeling

Chromatin is a highly dynamic structure of nucleosomes containing histone proteins that wrap around a stretch of DNA to assist in the folding of DNA inside the nucleus (Kornberg, 1974; Noll, 1974; Maclean and Hilder, 1977; Pennisi, 2003). Nucleosome occupancy is not homogenous across the genome, and certain regions in the DNA stretch are devoid of nucleosomes and are called nucleosome-depleted regions (NDRs) (Segal et al., 2006). NDRs are transcriptionally active regions. They are more accessible to DNA-binding proteins and factors that regulate transcription and gene expression (Yen et al., 2012). The positioning of NDRs is regulated by histone modifications, such as acetylation and methylation, and defines its accessibility to the binding of key transcriptional factors (Bannister and Kouzarides, 2011; Venkatesh and Workman, 2015). Hence, nucleosome positioning that maintains chromatin architecture specifies the epigenetic status of the cell, and this mechanism is also associated with the formation and stabilization of G-quadruplex structures. However, the implications of G4s in genomic DNA for chromatin remodeling and epigenetic programming have not been considered and explored until recently.

Epigenetic mechanisms, such as DNA methylation and histone acetylation and deacetylation, re-modulate chromatin architecture, which are dysregulated in AD (Delgado-Morales et al., 2017; Bano et al., 2021; Santana et al., 2023). Epigenetic profiles can vary throughout an individual’s lifetime, especially during the aging process, and pathological conditions, such as AD and factors such as stress and diet affect epigenetic expressions and neuropathology. G4 structures could play a prominent role in contributing to AD pathology.

By using G4-specific antibody chromatin immunoprecipitation (ChIP)–sequencing analysis, the Balasubramanian lab showed that the majority of G4s are localized in NDRs and associated with active transcription, indicating that chromatin remodeling programs G4-DNA formation (Chambers et al., 2015; Hansel-Hertsch et al., 2016; Hansel-Hertsch et al., 2018). The chromatin opening activates transcription, creates suitable conditions for the G4 formation, and can drive or facilitate epigenetic changes and reorganization (Sekibo and Fox, 2017; Zheng et al., 2017). It would be very interesting to determine the dynamic changes in G4s and G4-regulating proteins in the context of pathology associated with AD and AD–related dementias (ADRDs). Modulating changes associated with AD pathology can potentially benefit designing strategies to overcome disease manifestation. It is unclear if there are any cell-specific and tissue-specific changes in G4 homeostasis, and addressing their roles in specific cell types in the central nervous system will be important to decipher mechanisms contributing to pathology associated with aging and AD.

The role of G4s in chromatin remodeling was first explained by a study on REV1, a DNA repair protein that ensures DNA replication during DNA damage. The cells with mutant REV1 display delayed or compromised DNA replication, specifically at G4-forming sequences (Sarkies et al., 2010). The inability of the G4 structures to resolve in the REV1 mutant cells leads to replication stalling along with epigenetic changes. The expression of the p-globin locus was increased and associated with the loss of histone modifications along with the loss of H3K9 methylation leading to transcriptional activation (Sarkies et al., 2010). This study explains that G4-DNA formation resulted in repeated loading of newly synthesized histones that led to permanent loss of repressive epigenetic marks. Moreover, a study from the Balasubramanian lab explains the relationship between G4s and the incorporation of epigenetic marks. Unresolved G4 structures in REV1 mutants in chicken DT40 cells result in the loss of distinct histone marks promoting transcription (Schiavone et al., 2014). G4-DNA structures are co-localized with histone modifications in NDR euchromatin (Komurkova et al., 2021). These studies revealed the importance of G4-DNA structures in modifying the histone code, shaping the chromatin architecture, and therefore modulating the epigenetic landscape.

Histone modifications have been associated with synaptic plasticity, learning, and memory, and dysregulation of these processes is present in mouse models of aging and AD (Nativio et al., 2018). Loss of heterochromatin is coupled with reduced nucleosome occupancy during aging, resulting in the loss of transcriptional silencing and contributing to age-associated genomic instability. This process is strongly associated with accelerated aging, leading to reduced lifespan (Larson et al., 2012; Pal and Tyler, 2016; Sidler et al., 2017; Yi and Kim, 2020). Although there is no cure for AD, it is still possible to dwell on and design new strategies, and considering the novel regulatory functions of G4s in modulating the histone code could be the next subject of research that could lead us to new avenues and novel mechanisms in AD pathology.

G4-DNA structures can be stabilized or destabilized by methylation, based on the G4 topology and positions of methylated (m)CpG densities (De and Michor, 2011; Lin et al., 2013a; Stevens and Kennedy, 2017). The strong interactions between DNA methyltransferases (DNMTs) and G4-DNA motifs have been confirmed *in vitro* (Cree et al., 2016). G4s from the promoter regions of the oncogene c-MYC have shown efficient binding affinities to *de novo* methyltransferases DNMT1, DNMT3A, and DNMT3B while being limited to no binding to non-G4 mutants (Cree et al., 2016). G4 ChIP-seq analysis from human leukemia cells have revealed that the majority of G4 motifs are found in the open chromatin, overlapped with CpG islands. Interestingly, G4-CpG overlaps were hypomethylated and proximal to DNMT1-binding sites that were detected by ChIP-seq (Mao et al., 2018). In this study, the authors proposed that G4s in CpG islands sequester DNMT1, inhibiting its activity and cooperating with transcription factors to protect the CpG islands from methylation (Mao et al., 2018). Thus, G4-DNA may have a role in the formation and maintenance of CpG methylation and in modulating chromatin dynamics. In summary, G4-DNA interacts with DNMTs and transcription factors and may contribute to histone modifications, chromatin relaxation, and nucleosome repositioning during replication and transcription (De and Michor, 2011; Lin et al., 2013a; Chambers et al., 2015; Cree et al., 2016; Mao et al., 2018). However, G4-DNA may help shape the chromatin structure by altering nucleosome positioning and histone modifications, and G4 structures may simply form at open chromatin sites as a consequence of DNA accessibility and negative supercoiling required for active transcription (Selvam et al., 2014). Therefore, future studies must evaluate the direct causation between G4-DNA formation and chromatin remodeling and identify regulators that modulate the epigenetic landscape.

Aging-associated epigenetic changes include histone modifications, DNA methylation, and chromatin remodeling. All of these may contribute to regulating the aging process and age-related diseases, such as AD and other dementias. The consequences of epigenetic changes during aging include replicative senescence, altered accessibility to transcription factors, leading to aberrant gene expression, and genomic instability. However, few studies have tried to establish the connection between G4 structures that modulate the epigenetic landscapes and that which may contribute to the aging process and the pathology associated with neurological disorders. Some have shown that methylation at the C5 position of cytosine (5 mC) within G4 motifs confers a high degree of stability to G4-DNA (Hardin et al., 1993). Methylated cytosines at dCGG repeat within G4 motifs, and expansion of dCGG repeats are associated with the downregulation of the *FMR1* gene in fragile X mental retardation syndrome (Liu et al., 2018). In addition, C5 methylation within the hexanucleotide repeat GGGGCC in the non-coding region of the C9orf72 locus imparts stabilization to G4 structures and thereby implicates methylation-dependent G4 stabilization in diseases ALS and FTD (Zamiri et al., 2015). In contrast to the notion that G4s influence DNA methylation at specific sites, genomic instability was observed where G4s failed to resolve in cells lacking G4 helicases, such as PIF1, FANCJ, and BLM (Opresko et al., 2005; Wu et al., 2008; Paeschke et al., 2013; Nguyen et al., 2014b; Dahan et al., 2018). These findings suggest that G4s alter the placement of modified histone proteins that pack chromatin, which is a hallmark of epigenetic regulation (Sarkies et al., 2010).

Epigenetic abnormalities are observed at the onset and during the progression of age-related diseases, such as AD (Cavalli and Heard, 2019). The global DNA methylation pattern decreases with aging and contributes to aging-associated heterochromatin loss, and some genomic regions are also characterized by age-related locus-specific hypermethylation (Xiao et al., 2019; Ferret et al., 2023). Nevertheless, much progress has been made in understanding the genetic basis of AD in which multiple loci have been discovered. The changes in histone acetylation, methylation, phosphorylation, and other epigenetic modifications have been observed in aging, AD, and ADRDs. The next big question is to elucidate whether these changes are G4 dependent or cause and initiate the pathology by modulating the G4s and G4 regulatory proteins. It is also crucial to determine if the metabolic alterations driven by epigenetic changes are the cause or consequence of dysregulation in G4 homeostasis.

### G4-DNA and transcription

Putative G4-DNA motifs are enriched at transcription start sites and promoter regions, thereby regulating transcription and gene expression (Agarwal et al., 2014; Marsico et al., 2019). G4-DNA structures allow and inhibit transcription by recruiting proteins, blocking polymerases and topoisomerase poisoning, and keeping the nascent strand in a single-stranded conformation by the formation of a G4 structure on the non-transcribed strand (Balasubramanian et al., 2011; Berroyer and Kim, 2020; Bossaert et al., 2021) (Figure 2B). The role of G4-DNA in regulating gene expression is well studied in yeast and cancer cells but is very limited in other cell types (Cogoi and Xodo, 2006; Lopez et al., 2017). Stabilizing G4-DNA results in reduced mRNA transcript levels in genes that contain G4-DNA motifs in their respective promoters, such as proto-oncogenes *KRAS* and *c-MYC* (Siddiqui-Jain et al., 2002; Gonzalez and Hurley, 2010). G4-DNA found in the *c-MYC* promoter functions as a silencer element and associates with many G4-DNA–binding proteins, such as hnRNPA1, Eef1A, and RPS20 (Gonzalez and Hurley, 2010). Nucleolin, a specific G4-DNA–binding protein, acts as a repressor of *c-MYC* transcription by binding and stabilizing the formation of G4-DNA in the active regions of the *c-MYC* promoter (Gonzalez et al., 2009; Cogoi et al., 2014). H-*ras*, a proto-oncogene, also contains G4-DNA in its promoter regions, and MAZ (Myc-associated zinc-finger protein) is recruited to the G4-DNA motifs, leading to the activation of H-*ras* (Moruno-Manchon et al., 2020)*.* These findings suggest that G4-DNA motifs act as a molecular switch, regulating the switching ON or OFF of gene expressions via structural changes. By contrast, co-transcriptional activator Sub1 (PC4 is the mammalian homolog of Sub1), which interacts with G4-DNA and G4 helicase PIF1, suppresses G4-mediated genomic instability by facilitating the recruitment of PIF1 helicase to co-transcriptionally formed G4-DNA structures (Cogoi and Xodo, 2006). We previously demonstrated that PC4 and Sub1 bind to G4-DNA forming a sequence within the *Atg7* gene that regulates autophagy (Johnson et al., 2010). The G4-DNA helicases WRN and BLM contain G4 motifs in their promoter regions, and their aberrant function due to altered transcription establishes a link between G4-DNA and gene expression. In addition, cells deficient with BLM show high rates of sister chromatid exchange sites of G4-DNA motifs in transcribed genes (Rodriguez et al., 2012; Nguyen et al., 2014b; van Wietmarschen et al., 2018).

**FIGURE 3:**
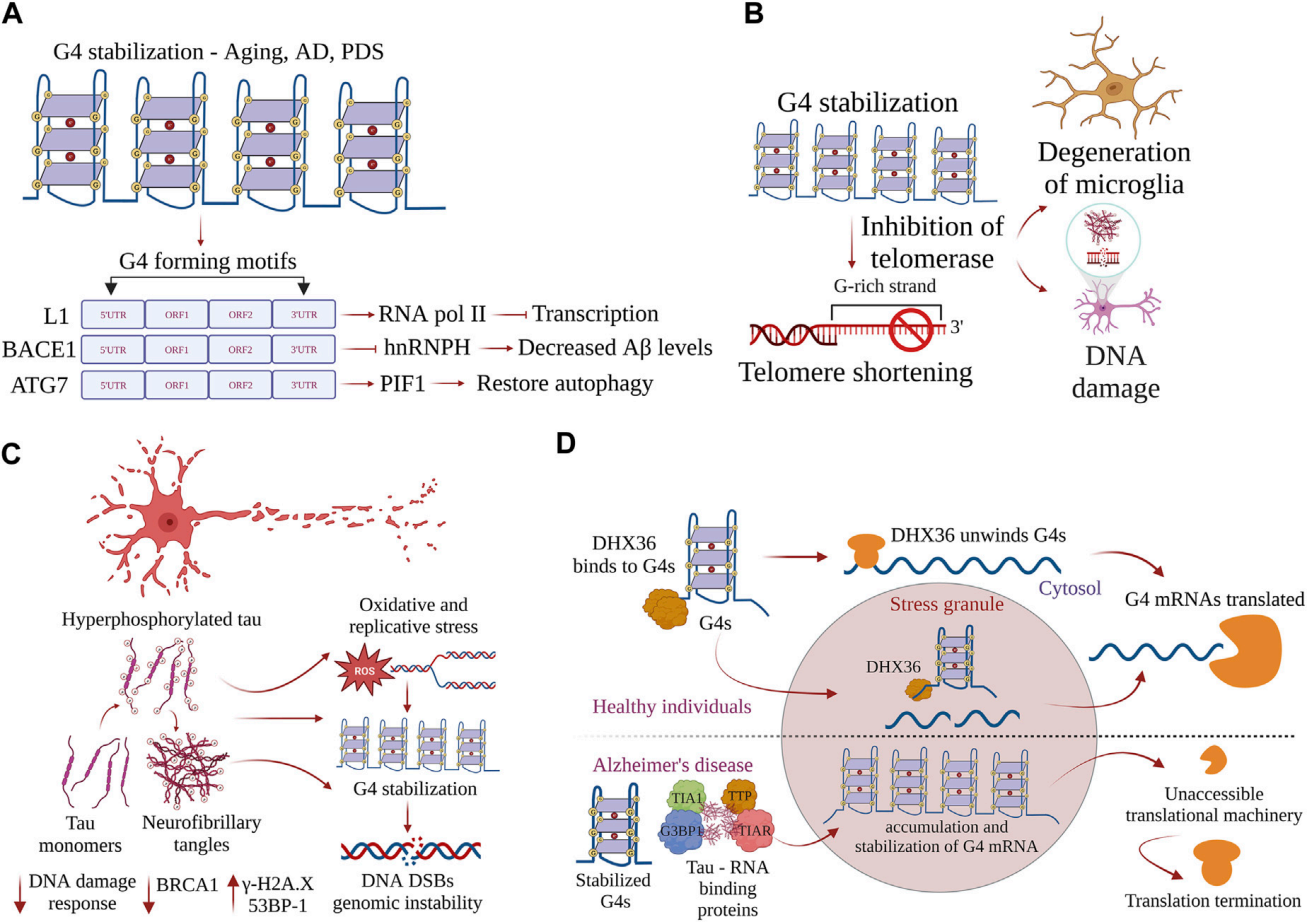
G-quadruplexes contribute to progeroid phenotypes in aging. G4s are overtly stabilized during aging and disrupt many physiological processes inside the cell. **(A)** Stabilization of G4 structures on the L1 sequences, BACE1, and ATG7 regulate the pathways driving AD pathogenesis, and restoring the homeostatic balance of G4 structures could provide new therapeutic avenues. **(B)** Accumulation of neurofibrillary tangles in AD-induced progression is exacerbated by stabilization of G4 structures, leading to DNA damage and causing genomic instability. *Postmortem* samples of AD brain samples show increases in γH2AX and 53BP1 and decreased DNA damage response. **(C)** G4 helicase DHX36 resolves G4 structures formed on the mRNA and prevents the accumulation of G4s in stress granules. In AD, G4 helicases lose function, G4 structures are overtly stabilized, and G4 binding proteins form complexes with tau aggregates. mRNAs with stabilized G4 structures become inaccessible, leading to termination of translation. **(D)** Stabilization of G4s inhibits telomerase activity, contributes to telomere shortening, and is associated with the accumulation of DNA damage and degeneration of glial cells.

Stabilization of G4-DNA structures by pharmacological means, such as pyridostatin (PDS), impedes the progression of the transcriptional machinery and affects gene expression (Scheibye-Knudsen et al., 2016; Miglietta et al., 2021). Many oncogenes and tumor suppressor genes, such as *SRC* and *c-MYC*, are downregulated by PDS treatment, and differences in transcriptional changes at G4-DNA motifs may be the result of direct G4-DNA stabilization or DNA damage–mediated transcriptional repression (Miglietta et al., 2021). We have shown that PDS alters autophagy in neurons and glial cells by reducing the expression of *Atg7* (Johnson et al., 2010; Noh et al., 2021). Moreover, we have shown that overexpression of PIF1 in the presence of PDS restores autophagy in cultured primary neurons and protects neurons from dying (Figure 3A). Helicase-dead mutant PIF1 could not rescue the effects of PDS-associated autophagy reduction, displaying the importance of G4-DNA structures in regulating the expression of survival genes (Johnson et al., 2010). Transcriptome analysis by RNA sequencing has revealed that G4-DNA stabilization by PDS activates the response of innate immune genes in human and murine cancer cells (Scheibye-Knudsen et al., 2016). Stabilizing G4 structures with PDS leads to accelerated aging in *C. elegans* (Lu et al., 2004). The transcription of many genes is altered in the aged brain (Lipinski et al., 2010; Masters et al., 2015). We demonstrated that aged mouse brains contain higher levels of G4-DNA than young mouse brains (Johnson et al., 2010). However, the regulatory functions of G4-DNA structures in modulating the transcriptome profile in neurons and glial cells are not known. G4-DNA could be regulating the expression of many essential genes crucial for cellular processes, such as autophagy, apoptosis, oxidative stress, protein misfolding, and mitochondrial damage, as it contains putative sequences that can fold into G4-DNA structures (Agarwal et al., 2014). The discovery of G4-DNA regulates gene expression, highlighting the fact that G4-DNA structures could be potential targets for disease treatment.

G4-DNA regulates the expression of several genes that are essential to pathways associated with AD. β-Amyloid precursor protein-cleaving enzyme 1 (BACE1) encodes a transmembrane protease that cleaves the amyloid precursor protein (APP) to generate the amyloid-beta (Aβ) peptide that misfolds and accumulates in AD (Fisette et al., 2012). The recruitment of heterogeneous nuclear ribonucleoprotein H (hnRNPH) to the G-rich region in the exon 3′ of BACE1 facilitates full-length functional proteins. However, the formation of G4-DNA within its G-rich region prevents hnRNPH recruitment and results in alternate splicing, producing shorter protein isoforms that lack proteolytic function (Hanna et al., 2021). Facilitating G4-mediated exon splicing by knocking down hnRNPH promotes the production of a shorter alternative BACE1 isoform that decreases Aβ production, suggesting G4-mediated splicing as a potential therapeutic strategy to mitigate the production of this AD-associated peptide (Hanna et al., 2021) (Figure 3A). A fascinating study recently demonstrated that G4-DNA structures in the active L1 sequences co-localize with RNAPII, which inhibits the overall transcription and affects splicing events, thereby affecting the neuronal gene expression in AD (Gyenis et al., 2023) (Figure 3A). Additionally, G4 motifs in the active L1 sequences control cell-cycle progression and apoptosis and potentially contribute to AD pathogenesis (Gyenis et al., 2023). Moreover, RNA polymerase stalling occurs more frequently in aging tissues than in young tissues (Miller et al., 2017). DNA damage reduces transcription efficiency, resulting in the dysregulation of pathways, such as autophagy, nutrient sensing, fatty acid metabolism, proteostasis, and immune function, that contribute to aging (Miller et al., 2017). Based on the role of G4-DNA in regulating the transcriptome, we hypothesize that G4-DNA structures play a significant role in controlling the expression of genes involved in pathology cascades associated with age-related neurological disorders.

Multiple genome-wide transcriptome changes are associated with aging and AD. Recently, a study analyzed the hippocampus and two regions of the cortex in 107 aged donors and identified sets of co-expressed genes correlated with pathological tau and inflammation markers (Marques-Coelho et al., 2021). Transcriptomics revealed robust and stereotyped gene expression patterns in spatial and temporal variations over the lifespan from development through adulthood into aging, and the aged brain displays greater variability in its transcription profile than do younger brains. Many gene expression studies conducted in younger cohorts have revealed dysfunction related to dementia phenotypes in a variety of biological pathways, such as energy metabolism, neuroinflammation, axon–myelin interactions, synaptic transmission, protein misfolding, and transcription factors. However, it is still unclear whether robust relationships between transcriptional changes and disease pathology or cognition deficits extend to older individuals (Marques-Coelho et al., 2021). In addition, gene expression analysis with RNA sequencing data from *postmortem* brain samples have found that alterations in the transcription profile are prominent in the temporal lobe—which is affected more in the early stages of AD pathogenesis—than in the frontal lobe (Hernandez et al., 2011). These discoveries are very interesting and raise important questions if there are any region-specific regulations of amyloid pathology in AD brains. It is still uncertain what determines the early transcriptional changes that are before β-amyloid and tau accumulation in AD brains. Many genes that are manifested in AD pathology are regulated by G4 structures, and the extent to which they contribute to disease pathology remains obscure. We hypothesize that transcriptional dysregulation could be mediated by an imbalance in G4 homeostasis and may well precede tau and amyloid aggregation. It is also possible that helicases and regulatory proteins that control G4 dynamics could be altered and contribute to transcriptional dysregulation in AD.

Thus, gene expression changes in the AD brain may occur at the transcript level, and several genes encoding proteins for alternative splicing machinery may be altered in the AD brain. In addition, a large number of isoform switches have been associated with alternative transcription start sites, termination sites, exon skipping, and intron retention. These alterations in the transcription and alternative splicing are more prominent in the temporal lobe affected early in AD (Hernandez et al., 2011). Epigenetic changes regulate gene expression, and DNA methylation in the brain decreases with age (Yu et al., 2015). Genes implicated in AD (e.g., *BDNF*, *MAPT*, *ANK1*, *SORL1*, *SIRT1*, and *APP)* show differential methylation patterns in individuals with AD and controls (De Jager et al., 2014; Mano et al., 2017). The DNA repair protein BRCA1 is hypomethylated in AD, and elevated levels of BRCA1 localize to the cytosol, co-aggregate with insoluble tau, and affect neurite and spine morphology (Pellegrini et al., 2021). In addition, epigenetic age acceleration is heritable in AD and associated with neuropathological protein accumulation and cognitive decline (Schaffitzel et al., 2001; Levine et al., 2015). Therefore, understanding the relationships among DNA methylation, aging, longevity, and age-related diseases may hold promise to predict the phenotypes and design strategies to combat the disease. Research pertaining to possible mechanisms driving the G4-dependent epigenetic and gene expression alterations in neurological disorders is limited. With the diverse physiological roles of G4s and G4 helicases in shaping the genome architecture, it is imperative to predict that G4 structures regulate a wide myriad of functions in disease-associated pathways. Many age-related neurodegenerative diseases share common downstream pathological cascades, and it is a challenge to design therapeutics that target specific pathways or a gene or a protein without showing any adverse off-target effects. Therefore, understanding the G4 dynamics in the aging process could provide us with novel mechanisms, and in the future, these G4s could be used as new molecular biomarkers to predict age-related neurological disorders.

### G4 structures at telomeres

Telomeres are a series of repetitive base-pair sequences at the chromosome ends that facilitate their replication. Telomeric DNA possesses a G-rich consensus sequence (TTAGGG)_n_ with a double-stranded portion of several kilobases and a 3′ overhang of a few hundred bases and has the propensity to fold into G4-DNA structures (Figure 2C). The formation of G4-DNA motifs was identified by diverse G4-specific antibodies, such as Sty3, Sty49, BG4, and D1 (Biffi et al., 2013; Kang et al., 2016; Liu et al., 2016; Javadekar et al., 2020). G4-DNA structures at the telomeres challenge the replication machinery and may form a barrier for replication forks, potentially leading to telomere instability. In cells lacking FANCJ, G4-DNA structures are stabilized and impede the progression of DNA polymerase, stalling replication and thereby causing DNA DSBs. Unresolved G4-DNA inhibits telomerase activity causing telomere shortening (Drosopoulos et al., 2015). These physiological events are vital in dividing brain cells, such as astrocytes and microglia, and dysfunctional telomerase activity could lead to serious deleterious effects in brain processes. Many G4 helicases (e.g., PIF1, WRN, BLM, RTEL1, and DNA2) exhibit telomere G4-DNA unwinding activity. Among them, PIF1 is the best characterized, whereas PIF1-deficient cells slow down the progression of DNA replication and induce DNA DSBs (Paeschke et al., 2013; Drosopoulos et al., 2015). BLM and WRN are recruited by shelterin proteins, and their deficiency results in the formation of G4-DNA structures in cells, especially at telomeres (Vannier et al., 2013). G4 helicase RTEL1 resolves G4-DNA in an ATPase-dependent manner, and cells deficient in RTEL1 display G4-DNA stability that dramatically enhances telomere fragility (Vannier et al., 2012). Disruption of the CST complex at telomeric G4-DNA motifs results in disrupted telomere replication and leads to telomere shortening (Masuda-Sasa et al., 2008; Zhang et al., 2019a). Mammalian DNA2 helicase is localized at telomeres, and DNA2 deficiency in mouse cells results in defects in telomere replication that are enhanced by G4-DNA stabilization (Takahama et al., 2013).

Chromatin homeostasis at telomeres and sub-telomeric regions depends on a telomeric repeat DNA that forms a G4-RNA (TERRA) structure that functions as a protein docking scaffold (Azzalin et al., 2007). TERRAs contain long non-coding RNA and telomeric transcripts (Arora and Maiti, 2009; Deng et al., 2009) and are the key to maintaining the telomeric structures via interactions with telomere-associated proteins, such as TRF2. TRF2 has a high affinity to TERRAs and relies on the formation of G4-RNA structures in TERRAs. G4-RNA–mutated TERRA repeats do not bind to TRF2 and impede the telomere length (Riou et al., 2002). The activity of telomerase is also influenced by the resolving G4s at the 5′-ends of the RNA component of telomerase by DHX36 (Booy et al., 2012). Telomere extension is prevented by stabilizing G4-RNA, leading to cellular senescence aiding the role of G4s in telomere maintenance (Sexton and Collins, 2011). DHX36 resolves G4-RNA in the RNA component of the telomerase, thereby enabling the formation of a stem-loop structure, which is the key for reverse transcription by telomerase (Booy et al., 2012). The siRNA-mediated downregulation of DHX36 compromises telomerase function by reducing the telomere length (Nguyen et al., 2017). The ATRX (alpha-thalassemia X-linked intellectual disability) protein prevents the formation of G4 structures within the R-loops to prevent replication stalling and maintain the telomere length (Wang et al., 2021).

Distinct from G4 helicases, some specialized G4BPs unwind G4 structures, and they do not require energy by ATP hydrolysis and unfold G4 structures by passive binding (Ray et al., 2013). RPA is a single-stranded DNA-binding protein that disrupts G4-DNA in the 5′→3′ direction and is involved in telomere maintenance (Ray et al., 2014; Safa et al., 2016; Chaires et al., 2020). POT1 (protection of telomere 1) is the key for telomere integrity by binding to the 3′-end overhang to prevent G4-DNA formation. POT1 specifically unwinds telomeric G4-DNA but no other G4 structures (Flanary et al., 2007). Upon G4-DNA formation at telomeres, the ability of POT1 to trap G4 structures outcompetes the ability of RPA to resolve G4-DNA. This action displays the protective function of POT1 at telomeres (Chaires et al., 2020).

Shorter telomeres are associated with the progression of AD. Telomere shortening contributes to the degeneration of microglia, an event that may alter AD pathogenesis (Rolyan et al., 2011) (Figure 3B). It reduces amyloid pathology and improves cognitive impairment by reducing the activation of microglia in aging APP23 transgenic mice (Tomita et al., 2018). Telomere shortening is also associated with the accumulation of DNA damage in the aging brain (Tomita et al., 2018). Telomeres in neurons remain stable throughout life, but those in the glial cells become significantly shorter with aging (Fani et al., 2020) (Figure 3B). Telomere shortening is also associated with reduced adult neurogenesis in the dentate gyrus, a fact that impairs the maintenance of neurons in aged late telomerase-deficient mice (Tomita et al., 2018). Importantly, shorter and considerably longer telomere lengths are significantly linked with an increased risk of dementia, especially AD (Spilsbury et al., 2015). The potential biological link between telomere length and a higher risk of AD has to be critically ascertained. By contrast, mice lacking telomerase reverse transcriptase (TERT) in neurons display shorter telomeres and increased oxidative damage in response to tau aggregates, demonstrating that shorter telomeres exacerbate pathological progression (De Meyer et al., 2018). These findings pose many questions about the crucial role of G4s in telomere maintenance. Future reports linking telomere length and disease progression are expected to provide important insights into AD pathophysiology.

The loss of telomere repeats in human cells with age differs widely between cells and tissues, and the mechanisms that regulate the telomere length in age-related neurological disorders are limited. It remains uncertain whether age-related telomere shortening is a cause or merely a consequence of aging-associated diseases (Gorgoulis et al., 2019). Moreover, telomere shortening is inadequate to explain the aging process in non-proliferating, quiescent, or terminally differentiated neuronal cells. There could be G4-dependent intrinsic molecular mechanisms that contribute to dysregulation in neuronal functions. With the enrichment of G4 motifs in telomeres, it would be interesting to determine to what extent G4s and G4 helicases regulate telomere homeostasis in aging and AD pathology.

In the broader context of the aging process, the notion that telomeric DNA is the only indispensable component of the cell makes a strong argument for an apical role of DNA integrity in the aging process. Moreover, in addition to the complex mechanisms involved in the repair of DNA damage, telomeric DNA is hypersensitive to oxidative damage that induces and accelerates telomere shortening (Figure 3B). The fact that G4 structures are more prone to oxidative damage creates a sustainable query on how G4 structures can regulate the telomere length during aging. The most prevalent hypothesis on telomere shortening and aging does not depend solely on telomerase dysfunction. Telomere dysfunction–activated DNA damage responses that cause cellular senescence may facilitate the age-related loss of tissue functions (Puget et al., 2019; Di Micco et al., 2021). Nevertheless, cellular senescence may occur independently of telomere dysfunction, and the differential contribution of telomere-dependent and telomere-independent mechanisms of senescence to aging and age-related neurological disorders in glia and other dividing cells remains an active area of investigation. Telomere dysfunction may not be the only cause of senescence and the aging process. The pathways that engage telomeric DNA damage responses may also be important. It may be deleterious to globally inhibit or blunt the pathways of DNA damage signaling and repair, the opportunities may lie in the selective DNA damage response ablation at dysfunctional telomeres. The discovery of G4-DNA and G4-RNA structures and their roles in DNA damage responses makes these secondary structures attractive targets for potential therapeutic interventions. Finally, in future, researchers should focus on delineating the G4-dependent molecular mechanisms that regulate telomere biology and on ascertaining and broadening the impact of G4 structures in telomere dysfunction in age-related neurological disorders.

### G4-DNA and DNA damage

In recent years, research efforts have focused on understanding the role of G4-DNA structures in inducing DNA damage in brain cells. In neuronal cells, overly stable G4-DNA stalls DNA polymerase during transcription (Thomas et al., 2008). The action of endonucleases through a mechanism of transcription-coupled repair poisoning then leads to DNA damage (Iyama and Wilson, 2013). DNA DSBs are more dangerous for neurons than they are for dividing glial cells, as these can effectively repair DSBs by homologous recombination using sister chromatids (Lieber, 2010). To repair DNA DSBs, post-mitotic neurons rely on a non-homologous end-joining mechanism that depends on error-prone DNA polymerases (Alt and Schwer, 2018; Shanbhag et al., 2019). Similar to cancer cells, dividing brain cells may use G4-DNA to promote DNA damage via a replication-dependent mechanism (Miglietta et al., 2021).

DNA DSBs causing genome instability are associated with aging and AD (Suberbielle et al., 2013; Suberbielle et al., 2015; Thadathil et al., 2021). Immunostained sections of the *postmortem* brain of individuals with AD and mild cognitive impairment (MCI) show a significant increase in nuclear 53BP1 staining in the frontal cortex and CA1 regions (Suberbielle et al., 2015). MCI and AD patients have a greater proportion of γH2AX-positive foci in the frontal cortex than do the controls. In addition, human *postmortem* AD brains display significantly more DSBs and less DNA repair function in the hippocampus than do the non-demented controls (Asada-Utsugi et al., 2022) (Figure 3C). Interestingly, similar results have been documented in the hippocampus of the 5xFAD transgenic mice and cellular models of AD (Asada-Utsugi et al., 2022). Since aging and DNA DSBs are risk factors for AD progression, a recent study involving human *postmortem* AD samples has revealed that DSBs are decisive in tau pathology of AD and the error of DNA repair is linked to tauopathy (Vermeij et al., 2016). DSBs are more prominent and extensive in AD brains than in age-matched control brains. Immunohistochemical staining of neurons, astrocytes, microglia, oligodendrocytes, and endothelial cells shows increased γH2AX-positive foci and phosphorylated tau in the cortex of AD patients (Vermeij et al., 2016) (Figure 3C). Thus, AD pathology is associated with the progressive accumulation of DNA DSBs and/or alteration in the expression of proteins of DNA repair pathways that may lead to cellular damage in AD. We hypothesize that an increase in DSBs in aging and AD brains may be due to the progressive accumulation and stabilization of G4-DNA structures. Additional studies are required to ascertain and unravel the molecular mechanisms of G4-DNA–associated DNA damage and genome instability in aging and AD.

The therapeutic strategy of activating the DNA damage response (DDR) is critical for preventing cancer; however, the chronic activation of DDR is thought to facilitate the accumulation of senescent cells and inflammation during the aging process. How does DNA damage mechanistically drive the aging process? Many mechanisms have been proposed, such as restricting transcription (Vijg, 2014), inducing mutagenesis (d'Adda di Fagagna et al., 2003), triggering senescence and apoptosis, and activating the signaling cascades (Kennedy et al., 2014). During the aging process, DNA damage occurs stochastically, and the type of DNA damage is influenced by intrinsic and extrinsic factors that involve methylases, histones, transcription and replication factors, and oxidizing agents, which include the dysregulated homeostasis of G4-DNA structures. Every factor that induces DNA damage that might drive the aging process is genetically determined via distinct cellular responses to DNA damage (Hudson et al., 1998). Mitochondrial DNA damage and defects in base excision repair can adversely affect neuronal functions, thus increasing the risk of accelerating the aging process and contributing to neurodegenerative phenotypes (Barja and Herrero, 2000; de Souza-Pinto et al., 2008; Lillenes et al., 2013). Oxidative DNA damage to neuronal cells may be one of the most important components of aging as base-excision repair is important in preventing AD (Sai et al., 1992; Canugovi et al., 2013). The human brain consumes oxygen at a high rate, exposing the neurons to the associated activated reactive oxygen species (ROS) by-products. If antioxidants are limited or depleted in the brain, the neuronal cells are more susceptible to ROS-induced DNA damage (Hirano et al., 1996; Kaneko et al., 1996; Fleming and Burrows, 2020). The oxidative DNA damage is increased in genes that are rich in G-rich sequences and have the propensity to form G4-DNA motifs (Brosh and Bohr, 2007; Bielskute et al., 2021; Wu et al., 2023). In humans, premature aging and early death are characteristics of rare heritable diseases that are linked to defects in DNA repair or the processing of DNA damage that involves the dysregulation of RecQ helicases that regulate G4 structures (Liano et al., 2021).

Human diseases of premature aging include Cockayne syndrome, Werner syndrome, and Hutchinson–Gilford progeria syndrome. Cockayne syndrome is a premature aging disorder associated with specific defects in DNA repair and transcription. It is linked to G4-DNA structures, and the endogenous protein Cockayne syndrome B (CSB) selectively binds to G4-DNA structures and loss of its binding affinity elicits premature aging phenotypes (Stevnsner et al., 2008). CSB has a role in transcription, base-excision DNA repair, and maintenance of mitochondrial function (Kikin et al., 2008; Osenbroch et al., 2009; Aamann et al., 2010; Scheibye-Knudsen et al., 2012). More intensive studies must be carried out to establish the relationship between how G4 structures modulate the DNA repair pathways in the aging process and age-associated neurological disorders, such as AD.

### G4-RNA in splicing

G4-RNAs are implicated in mRNA splicing, and genome-wide analysis of alternatively spliced transcripts has revealed over 3 million putative G4-RNA–forming motifs that map to approximately 30,000 mammalian genes (Kostadinov et al., 2006; Didiot et al., 2008). G4-RNA motifs assembled in the proximity of splice sites may directly affect the binding of regulatory RNA-binding proteins modulating alternate splicing events that impact spliceosome assembly. G4-RNA structures are associated with exon and intron splicing enhancers and silencers. Two G4-RNA motifs are present within FMRP-binding sites on its pre-mRNA (*FMR1*) which gives rise to different FRMP isoforms that include longer and shorter isoforms (Gomez et al., 2004). The FMRP-binding site is a potent exon splicing enhancer and acts as a control regulatory element that modulates alternate splicing events in response to intracellular levels of FMRP (Gomez et al., 2004). Mutations in the FMRP-binding site affect its ability to form a G4-RNA structure and decrease FMRP binding, thus ablating exonic splicing enhancer activity and changing the splicing pattern of *FMR1* pre-mRNA (Gomez et al., 2004). G4-RNA structures found in intron 6 of the human telomerase (hTERT) serve as an intronic splicing silencer, and stabilized G4-RNA structures impair hTERT splicing (Vo et al., 2022). In *TP53* pre-mRNA, G4-RNA motifs on the intron 3 stimulate splicing of intron 2 acting as an intronic splicing enhancer resulting in the differential expression of transcripts with distinct p53 isoforms (Marcel et al., 2011). Moreover, G4-RNA structures promote exon inclusion, especially in the context of the CD44 gene, thereby regulating the switch between epithelial and mesenchymal states, which is crucial for tumor metastasis (Huang et al., 2017). G4-RNA structures within the I-8 element of the *CD44* gene promote alternative splicing and lead to the formation of the epithelial-specific CD44v isoform. The heterogeneous nuclear ribonucleoprotein F (hnRNPF) binding to the G4-RNA motif promotes the inclusion of CD44 variable exon v8, resulting in the inhibition of epithelial–mesenchymal transition (EMT) and EMT-associated cell migration and invasion. hnRNPF regulates G4-RNA–associated alternative splicing across the transcriptome, connecting G4 structures to EMT, and highlights the importance of G4s in regulating RNA splicing and gene expression. The RNA-binding protein HNRNPH1 interacts with G4-RNA sequences and regulates RNA processing. HNRNPH1 binds to the G-rich sequences and destabilizes the G4-RNA structures formed by EWSR1-exon 8 and mediates its exclusion from the oncogenic EWS-FL1 transcripts in Ewing sarcomas (Georgakopoulos-Soares et al., 2022). In a recent study, G4-RNA motifs were enriched near splice junctions and strongly associated with skipped exons in depolarized mice and human neurons (Harries et al., 2011). Exon–intron junctions in humans have displayed G4-RNA motifs, both at the 3′ and 5′-UTRs, and 31% of human genes have shown G4 motifs at least near one splice site within a distance of 100 bp. For example, HNRNPK and HNRNPU have shown high binding affinity to the G4-RNA motif around splice sites and have directly affected the regulation of G4-mediated alternative splicing (Harries et al., 2011). These findings indicate an evolutionarily conserved splicing regulatory mechanism where G4-RNA structures play a crucial role. The studies have provided a genome-wide characterization of the impact of G4 structures on alternative splicing, an area that has not been well explored. Overall, these findings emphasize the importance of splicing events in cell growth, differentiation, and responses to environmental changes and pathogens, with crucial implications for understanding splice regulation and the role of G4-RNA structures in gene regulation in human health and age-related diseases.

Splicing has a vital role in the aging process, and dysregulation results in abnormal protein production or mRNA nonsense-mediated decay. Moreover, changes in the splicing factors also affect splicing outcomes. Age-associated splicing dysregulation has been observed in diseases and in aging itself. Changes in the splicing factor expression and the occurrence of alternative splicing events have been detected in aging-related tissues, such as the brain, blood, senescent fibroblasts, and endothelial cells and changes have occurred in genes involved in metabolism and DNA repair (Tollervey et al., 2011; Holly et al., 2013; Lee et al., 2016). More changes in exon splicing events have been observed in the hippocampus of aged mice than in young ones, and similar intron retention patterns have been identified in the human cerebellum and prefrontal cortex (Stilling et al., 2014; Adusumalli et al., 2019). Since aging is one of the major risk factors for AD, genome-wide alternative splicing changes have been discovered with advanced aging in the brains of mice and humans (Mazin et al., 2013; Lee et al., 2016; Wang et al., 2018). In addition, RNA-sequencing data analysis conducted across 48 different human tissues has identified 49,869 tissue-specific age-associated splicing events, and these splicing changes broadly correspond to genes involved in aging, such as in DNA damage, repair, and apoptosis (Fay et al., 2017a). These findings suggest that splicing serves as a biomarker for biological aging and life expectancy and as a potential therapeutic opportunity to combat age-associated neurological disorders. The effects of the hallmarks of aging, such as defects in protein homeostasis, telomere attrition, and genomic instability, all affect RNA splicing due to their mechanistic interplay and in turn be affected by the loss of RNA homeostasis. However, the roles of G4-RNA and RNA-binding proteins in regulating the splicing events in disease-modifying pathways associated with aging and AD are not completely explored. Therefore, elucidating the role of G4 structures in splicing factors and downstream splicing targets in aging and longevity is now an active area of investigation. Furthermore, the challenge is to discriminate whether G4-mediated RNA splicing events seen with age are functionally adaptive responses to dynamic changes in cellular conditions or aberrant changes that induce dysfunction.

### G4-RNA in translation

G4-RNA motifs are found in protein-coding regions and are implicated in mRNA processing, RNA translocation, and regulation of translation (Kharel et al., 2020a; Kharel et al., 2020b) (Figure 2D). G4-RNA structures function as translational repressors and modulate mRNA polyadenylation and splicing (Stults et al., 2009; Kharel et al., 2020b). Ribosomal RNA (rRNA), the most abundant cellular RNA, forms highly stable G4s *in vitro* and is one of the most commonly rearranged chromosomal regions in solid tumors (Penev et al., 2019; Varshney et al., 2021). Many helicases of the DEAH-box family interact with G4-RNA (e.g., DDX3X, DHX36, and eIF4A). DDX3X has many binding sites, primarily in the 5′-UTRs that correspond to G4-RNA motifs and mRNAs encoding ribosomal proteins, and the knockdown of DDX3X suppresses the synthesis of ribosomal proteins (Nie et al., 2015). DHX36 has a high specificity for G4-RNA and was originally described as an RNA-associating protein that binds to the AU-rich elements in the 3′ regions of the mRNAs that facilitate its degradation (Tran et al., 2004). DHX36 regulates *NKX2-5* mRNA translation by unwinding G4-RNA structures. Upon DHX36 deletion, there is a reduction in the levels of NKX2-5 that implies repression of *NKX2-5* translation (Liu et al., 2021a). DHX36 also plays a role in C9orf72 repeat-associated non-AUG translation in ALS (Newman et al., 2017). DHX36 mediates pre-mRNA 3′-end processing of p53 by specifically unwinding parallel G4-RNA to maintain p53 levels, thereby conferring its role in DNA damage responses (Benhalevy et al., 2017). Translation initiation factor eIF4A unwinds G4-RNA structures in the 5′-UTR of mRNAs encoding many transcription factors, oncogenes, and epigenetic regulators (Wolfe et al., 2014). Inhibiting eIF4A activity results in reduced translational efficiency and translationally repressed mRNAs are enriched in G4-RNA motifs, especially in 5′-UTR (Wolfe et al., 2014). CNBP (human CCHC-type zinc-finger nucleic acid–binding protein) selectively binds to G-rich regions in mature mRNAs and can form G4-RNA structures. Depletion of CNBP decreases the translational efficiency of CNBP targets, indicating the key role of CNBP in regulating the translation process by preventing the formation of G4-RNA structures (Guenette et al., 2017). The APP mRNA transcript is transported to neuronal dendrites, where it is important in synapse formation (Westmark and Malter, 2007). APP translation is repressed by FMRP binding to G-quadruplexes in the APP coding region (Morris et al., 2010).

G4-RNA motifs can also affect mRNA translation by modulating the binding and localization of some translation-related factors to mRNAs. Human vascular endothelial growth factor mRNA has a G4-RNA motif in one of its IRES sites. When the G4-RNA motif in the IRES site is mutated, the translation initiation activity of IRES is completely suppressed (Bhattacharyya et al., 2015). The G4-RNA motif has a binding affinity for VEGF IRES and is required to recruit the 40S ribosomal subunit. Deletion of the G4-RNA motif results in decreased binding affinity, implying the essential role of the G4-RNA motif in translational regulation (Lee et al., 2020). Apart from their presence in open reading frames, G4s are abundant in the 5′ and 3′-UTR regions and regulate the translation of mRNA (Lammich et al., 2011). Many regulatory genes (e.g., *Adam10*, *Tgfb2*, *Fmr1*, *Nrf2*, *Snca*, and *Nrxn2*) contain G4-RNA motifs in their 5′-UTRs that control translation levels (Khateb et al., 2007; Westmark and Malter, 2012; Agarwala et al., 2013; Koukouraki and Doxakis, 2016; Lee et al., 2017; Ding et al., 2020). The 3′-UTR of APP contains multiple regulatory sequences that affect the stability and translation of the APP transcripts. The G4-RNA structures in the 3′-UTR of mRNA negatively regulate the expression of APP post-transcriptionally and may contribute to AD pathogenesis (Crenshaw et al., 2015). Increased expression of APP resulting from the loss of regulation by the G4-RNA leads to elevated Aβ levels (Kelmer Sacramento et al., 2020).

The correlation between mRNA and protein levels is progressively decoupled during aging and is observed in aged killfish, macaque, and human brains (Wei et al., 2015; Hu et al., 2018). Aging affects the rate of protein translation, and global protein translation is generally high during early adulthood and significantly drops with age in yeast (Depuydt et al., 2016), *C. elegans* (Webster and Webster, 1979), *Drosophila* (Hrachovec, 1969; Bailey and Webster, 1984), rodents (Young et al., 1975; Dwyer et al., 1980; Ekstrom et al., 1980; Blazejowski and Webster, 1983; Chocron et al., 2022), and humans (Rooyackers et al., 1996; Ravi et al., 2018). Age-related decline in protein translation has been observed in a wide range of cellular fractions, tissues, and organs, such as in the brain, heart, liver, muscle, kidney, and intestine. There is only one study that has reported increased protein translation with age in the heart tissue of 4- to 10-month-old mice (Vargas and Castaneda, 1983). The exact molecular mechanisms underlying age-related decline in protein translation are not known; however, many studies have suggested that decreased activity and levels of eIFs (eukaryotic initiation factors) impair the initiation step and contribute to an age-dependent decline in overall protein synthesis. The levels and activity of eIF2 in promoting ternary complex formation have been shown to decline with age in multiple tissues of rodents, such as in the brain, liver, kidneys, and spleen (Cales et al., 1986; Kimball et al., 1992; Luchessi et al., 2008). In addition, the activity and levels of eIF2B required for replenishing eIF2 activity also reduce with age in the brains and livers of rats (Luchessi et al., 2008). eIF5 that promotes the formation of the 43S pre-initiation complex also decreases during aging in multiple areas of the rat brain (Blazejowski and Webster, 1984). Apart from a decrease in activity of proteins regulating translation initiation, the elongation step and ribosome loading to mRNA are also compromised with aging (Scheuner et al., 2005; Stein et al., 2022). As discussed earlier in this section, G4-RNA and G4 helicases regulate the function of many proteins that modulate protein synthesis under normal and diseased conditions. Nevertheless, the exact molecular mechanisms on how G4 structures control the levels and processing of mRNA transcripts in aging and AD have not been fully explored. We have reported that age-dependent increase and stabilization of G4 structures impair cognitive ability in mice and reduce the activity of autophagy during aging (Johnson et al., 2010). These findings correlate with reduced levels of translation during aging, and G4 structures can regulate the levels of protein in aging. As protein translation decreases with age in humans and other mammals, one would expect that it may have detrimental effects on cell growth and survival. However, life-long reductions in protein translation slow down the aging process, ameliorate cellular senescence, and prolong lifespan in many age-related disorders (Hamilton et al., 2005; Pan et al., 2007; Syntichaki et al., 2007; Selman et al., 2009; Tain et al., 2009; Rogers et al., 2011; Martin et al., 2014; Takauji et al., 2016; Ren et al., 2019). In *C. elegans,* knockdown of translation initiation factors (e.g., eIF4E, eIF4G, eIF1, eIF2, eIF2B, eIF4A, and eIF5A) has significantly improved the lifespan (Chen et al., 2007; Tohyama et al., 2008; Ghosh et al., 2020). Pharmacological inhibition of protein translation using cycloheximide that targets the elongation phase of translation has abolished senescent phenotypes in human fibroblast cells and also improved lifespan in *C. elegans.* Considering the information discussed above, it is suggested that overactivation of protein translation may contribute to AD pathogenesis. The cytoplasmic FMR1-interacting protein (CYFIP) that downregulates translation by blocking eIF4E-eIF4G interactions is seen reduced in the *postmortem* brains of AD patients, and CYFIP reduction leads to increased AD pathology in mice (Min et al., 2015; Tiwari et al., 2016). Moreover, tau K174 acetylation in the brains of AD patients and mice have shown increased protein translation by causing nucleolar expansion (Caccamo et al., 2015; Portillo et al., 2021). Reducing protein translation by inhibiting S6K signaling improved spatial memory and restored synaptic activity in an AD mouse model (Collie et al., 2010). In summary, research so far has provided us with two conflicting results and has postulated that reduction in protein translation accelerates the aging process and that life-long reduced protein translation robustly improves health and lifespan. This paradox may be explained by assuming that age-related decline in protein synthesis is a passive byproduct of aging and that proteostasis imbalance declines with age. So, to balance proteotoxic stress, protein translation may have been repressed as an adaptive response. Here, can we speculate if the stabilization of G4-RNA is responsible for translation repression during the aging process? Does the expression of G4-RNA–regulating proteins differ in AD pathological conditions? Nevertheless, to validate these mechanisms, more extensive research is required, and investigating the role of G4s and G4 helicases could be the next exciting area into how protein synthesis is regulated during aging.

### G4-RNA in non-coding RNA

G4-RNA is present in ncRNAs, especially long ncRNAs, TERC, TERRA, and microRNAs (Hirashima and Seimiya, 2015; Rouleau et al., 2018). Elevated levels of TERRA suppress vital innate immune genes, such as *STAT1*, *ISG15*, and *OAS3*, in cancer cell lines. Since TERRA has a higher propensity to form G4-RNA structures, innate immune gene expression changes are associated with G4-RNA formation (Matsumura et al., 2017). Inhibition of GSEC long ncRNA (G-quadruplex–forming sequence containing long ncRNA) transcription results in decreased mobility of colon cancer cells. GSEC long ncRNA binds to DHX36 and inhibits its helicase activity, thereby preventing G4-RNA unwinding. Thus, GSEC long ncRNA plays a significant role in the migration of colon cancer by suppressing the activity of DHX36 (Imperatore et al., 2020).

The ncRNAs have important roles in neural gene expression associated with neurological disorders. In neurons, miR-1299-3p regulates the expression of SORL1 (sortilin-related receptor) functioning as an apolipoprotein E receptor and affects APP processing and trafficking, specific tau interactions, and Aβ peptide dissolution (Rogaeva et al., 2007). Reduced expression levels of SORL1 protein in neuronal cells are associated with AD progression (Yamanaka et al., 2015). The precursor mir-1299-3p transcript contains G4 motifs that coexist in equilibrium with an extended hairpin structure and prevent the processing of the transcript into a mature miR form. The single-nucleotide polymorphism (SNP) variant re2291418 within the miR-1229-37 genomic sequence is associated with AD and destabilizes the G4-RNA structure in the precursor mir-1229-3p transcript (Rogaeva et al., 2007). In AD, long ncRNAs contribute to Aβ aggregation and dysregulated synaptic plasticity. Differentially expressed long ncRNAs, such as *SORL1-AS*, *LRP1-AS*, *BACE1-AS*, and *UCHL1-AS*, regulate gene expression and splicing of proteins involved in the production and trafficking of Aβ (Faghihi et al., 2008; Lian and Gallouzi, 2009; Ciarlo et al., 2013). It would be interesting to investigate the role of G4 motifs in long ncRNAs and how they contribute to Aβ aggregation. Thus, G4-RNA ncRNAs readily form and play important regulatory roles in aging and AD.

### G4-RNA in stress granule function

G4-RNA structures accumulate in the cytoplasm and participate in the formation of stress granules and disrupt mRNA translation (Kedersha et al., 2013). Stress granules are cytoplasmic aggregates of mRNA and ribonucleoproteins that regulate mRNA function, localization, and turnover (Kawane et al., 2014; Waris et al., 2014). G4-RNA formed from the damaged G-rich sequences provides a direct mechanism for cells to sense oxidative stress–induced DNA damage that leads to senescence (Byrd et al., 2016). In a proteomic screen to identify proteins associated with stress granules, the DHX36 helicase had been identified (Chalupníková et al., 2008). G4 helicase DHX36 binds to and resolves G4-RNA and can also be recruited to stress granules, likely playing a significant role in translation (Chalupníková et al., 2008) (Figure 3D). In addition to DHX36 associated with stress granules (DeJesus-Hernandez et al., 2011), other enriched proteins in the proteomic screen include TIA1 (T-cell intracellular antigen 1), ELAV1/HUR (embryonic lethal, abnormal vision, *Drosophila*)-like 1 (human antigen R), and YB-1/YBOX1 (Y-box–binding protein 1) that regulate translation and assemble into stress granules. The stress granule marker TIA1 co-localizes with G4-DNA–induced stress granules in the cytoplasm (Chalupníková et al., 2008). These studies have suggested a fundamental biological response to G4s and support that stress granules accumulate upon oxidative stress in the cytoplasm and promote cellular senescence. Expansion of hexanucleotide GGGGCC (G4C2) repeats in the non-coding regions of *C9ORF2* is the most common mutation associated with ALS and FTD (Renton et al., 2011; Fay et al., 2017b). Expression of RNA G4C2 promotes stress granule assembly in a repeat length in a G4-dependent manner, suggesting vital roles for RNA structures in the formation of stress granules (Ash et al., 2014).

In AD, pathological aggregation of tau may be initiated through regulated self-assembly of the core stress granule–associated RNA-binding proteins, such as TIA1, G3BP1 (Ras-GTPase–activating protein binding protein 1), TIAR (T-cell–restricted intracellular antigen 1), and TTP (Tristetraprolin), and TIA1 has been shown to interact and aggregate with hyper-phosphorylated tau in 8-month-old P301L mice (De Magis et al., 2019) (Figure 3D). G4s regulate the formation of stress granules and one of the characteristic features of AD-related pathology is the progressive accumulation of pathogenic stress granules, but the relationship between these two events has not been explored. It would be interesting to investigate the G4 landscapes in AD mouse models and the associated formation of stress granules.

### Anti-cancer therapy targeting G4-DNA may contribute to aging and dementia

G4 stabilization drives genome instability by introducing mutations, deletions, and stimulating recombination events (Lopes et al., 2011; Sauer and Paeschke, 2017; Puig Lombardi et al., 2019; Tan and Lan, 2020). In multiple forms of cancer, G4 formation and stabilization alter telomerase activity, inhibit replication, induce genome instability, and downregulate the expression of many proto-oncogenes responsible for tumor progression. Therefore, G4 structures are actively used as a therapeutic target to restrict tumor growth (Li et al., 2013; Bryan, 2019; Kim, 2019; Bryan, 2020; Carvalho et al., 2020). G4s are targeted pharmacologically by G4 ligands or by proteins that modulate G4 landscapes. Researchers have exploited this mechanism and designed strategies to modulate G4 structures with the idea to develop novel therapeutic drugs to fight against cancer (Li et al., 2013). More than 1,000 different G4 ligands have been developed, and their functions differ based on their specificity, binding surface properties, and cell permeability (Kim et al., 2002). The efficiency of these G4 ligands in modifying the G4 structures is not uniform: most of them are tested *in vitro*, and their potencies in the native physiological environment are still fiercely debated. Nevertheless, G4 ligands are widely accepted and include telomestatin, TMPyP4, PDS, RHPS4, and BRACO19, specifically binding to G4 structures over the DNA duplex (Izbicka et al., 1999; Gowan et al., 2001; Burger et al., 2005; Sun et al., 2005; Rodriguez et al., 2008).

In most cancers, promoters of the oncogenes accommodate more G4 motifs than do the promoters of regulatory or tumor suppressor genes (De and Michor, 2011). Therefore, many *in vitro* and *in vivo* experiments have proved that modifying or stabilizing G4 structures in promoter regions will reduce the expression of tumor-causing genes, such as VEGF (Dexheimer et al., 2006), KRAS (Siddiqui-Jain et al., 2002), BCL2 (Yang and Hurley, 2006), and MYC (Gonzalez and Hurley, 2010). Specifically, G4-mediated changes in the *MYC* transcription factor gene have been extensively investigated and are also upregulated in more than 70% of all cancers (Dang, 2012; Lin et al., 2012). Modifying G4 motifs in the *MYC* promoter drives oncogenesis by reducing the expression of MYC and thereby disrupting cell proliferation, migration, immune evasion, and metabolism, resulting in reduced tumor progression (Lin et al., 2012; Whitfield et al., 2017). One of the major drawbacks is the lack of direct inhibitors, and the current strategy only aims at targeting MYC expression (Kim et al., 1994). In 80%–90% of all cancers, the activity of telomerase is upregulated and facilitates cell division without telomere shortening (Moye et al., 2015). G4 structures at telomeres hinder telomerase binding, thereby blocking telomerase activity *in vitro* and *in vivo* (Zahler et al., 1991; Paeschke et al., 2005; Neidle, 2010; Paudel et al., 2020)*.* Thus, a working model shows that G4 stabilization at telomeres is used to restrict telomerase activity in tumor cells and prevent uncontrolled DNA replication, whereas telomerase is not expressed in somatic cells and remains unaffected (Tauchi et al., 2003). Many G4 ligands have been used to reduce tumor growth, such as telomestatin and RHPS4, which inhibit telomerase function by disrupting the telomere shelterin complex (Phatak et al., 2007; Zhu et al., 2012). Additionally, specific porphyrin derivatives, such as ZnP1 and TMPipEOPP, target telomeric G4s (Beniaminov et al., 2016; Moruno-Manchon et al., 2017). Stabilizing G4 motifs by G4 ligands causes DNA DSBs, pauses replication, induces micronuclei formation, and also restricts telomerase activity (Sun et al., 2005; Thomas et al., 2008; Lopes et al., 2011; Miglietta et al., 2021).

G4 ligands act via different mechanisms and most likely have multiple targets that are independent of tumorigenesis and cancer progression. The DNA-damaging agents, such as G4 ligands, are considered to be cytotoxic and drive genome instability that often leads to co-morbidities and makes disease even more lethal. Therefore, this raises some important questions about whether the burden of tumor has an impact on the outcome of G4-ligand treatment. What enduring effects does treatment with G4 ligands have on cancer patients? Changing the G4 landscapes leads to genome instability, does it also accelerate the aging process and lead to dementia? Reducing telomerase activity will suppress tumor growth and lead to cell death, but its reduced activity has been implicated in the progression of AD to early dementia (Sanders, 2010; Sexton and Collins, 2011). In cancer cells, stabilizing G4 motifs in the promoter regions has been beneficial in reducing the expression of oncogenes, but the effect of G4 ligands being global and not targeted will eventually lead to the suppression of expressions of critical survival genes and transcription factors, thereby having a seriously deleterious effect. Genomic instability that reduces gene expression and limits telomerase activity may be advantageous in cancerous cells to kill them, but in post-mitotic neurons, it will have a profound effect in conferring cellular senescence that may be detrimental and lead to neuronal damage. Neuronal cells cannot afford to hold and sustain these irreversible and irremediable changes incorporated by G4 ligands in the genome. To date, little is known about the risks of long-term treatment and how G4 landscapes change over time during treatments. The concerns addressed here are prominent and of high relevance to the fields of aging, dementia, and neurodegeneration and should be addressed in the near future.

## Conclusion and future directions

Studies on G4 structures, G4BPs, and various helicases have provided evidence that these molecules and structures regulate virtually all crucial cellular functions. Research on G4-DNA/RNA has recently sparked interest in understanding the molecular mechanisms of aging and age-related neurodegenerative disorders. Research is underway to understand if senescence and aging can be modified by genetic factors, especially by regulating the dynamics of G4-DNA/RNA by G4BP and helicases. In this review, we have discussed the relevance of G4 structures, associated proteins, and helicases in regulating mechanisms that could drive aging and related pathogenesis associated with AD. G4s, G4 helicases, and their synergistic interactions across the biology of aging and AD-associated pathways raise optimism that effective targeting of G4s may exert novel findings on how they drive the aging process. The transition of these G4-dependent cellular and molecular processes in aging and AD is complex and may not be linear. Many outstanding questions have to be addressed like how G4 dynamics are being regulated in different cell types in the brain. Somatic mutations, DNA repair, and DNA damage response could be differentially associated with Aβ and tau aggregation in AD, but how G4s and G4 helicases regulate these events has not been investigated. Activated microglia and astrocytes and neuroinflammation occur in aging and AD, and many proteins that control these mechanisms are differentially regulated; however, no data have explained the significance of G4 structures in these processes. Programmed cellular senescence, chromatin remodeling, and telomere attrition could not provide sufficient justification for the etiology of aging and AD, and investigating these mechanisms driven by G4s could open new horizons that can eventually be explored in delineating the pathology. To effectively combat aging and AD, pleiotropic drugs may be required to hit the right nodes of relevant G4-dependent pathways that are affected by aging or AD, and these could positively influence the outcome. Age-related decline in metabolic functions, neuroinflammation, epigenetic dysregulation, telomere dysfunction, and transcriptional aberrations may be the upstream causes of neuronal dysfunction and death, leading to pathological hallmarks, and have been the drug targets in AD. A better understanding and translation of the G4-dependent systemic, cellular, and molecular processes of aging and AD can help identify new strategies and therapeutic targets for drug discovery and development.
